# Frequency and component analysis of contaminants generated in preparation of anticancer agents using closed system drug transfer devices (CSTDs)

**DOI:** 10.1038/s41598-021-03780-0

**Published:** 2022-01-07

**Authors:** Satomi Sumikawa, Yoshihiro Yakushijin, Kenjiro Aogi, Takuya Yano, Hiroki, Hashimoto, Chiyuki Tsukui, Tadashi Noguchi, Taro Shiraishi, Yasuhiro Horikawa, Yasuo Yasuoka, Akihiro Tanaka, Noriaki Hidaka, Mamoru Tanaka

**Affiliations:** 1 Division of Pharmacy, Ehime University Hospital, Ehime, 7910295 Japan; 2 Department of Clinical Oncology, Ehime University Graduate School of Medicine, Shitsukawa, Toon, Ehime, 7910295 Japan; 3 Division of Clinical Research Promotion, NHO Shikoku Cancer Center, Ehime, 7910280 Japan; 4 Division of Pharmacy, Sumitomo-Besshi Hospital, Ehime, 7928543 Japan; 5 Department of Pharmacy, Matsuyama Red Cross Hospital, Ehime, 7908524 Japan; 6 Division of Pharmacy, Matsuyamashimin Hospital, Ehime, 7900067 Japan; 7 Department of Pharmacy, Saiseikai Imabari Hospital, Ehime, 7991592 Japan; 8 Department of Pharmacy, Shikoku Central Hospital of the Mutual aid Association of Public School teachers, Ehime, 7990193 Japan; 9 Jyuzen General Hospital, Ehime, 7928586 Japan; 10 Saiseikai Matsuyama Hospital, Ehime, 7918026 Japan; 11 Division of Pharmacy, Saiseikai Saijo Hospital, Ehime, 7930027 Japan

**Keywords:** Cancer, Cancer therapy

## Abstract

Occupational exposure of anticancer agents during their preparation has been recognized as a serious hazard. Closed system drug transfer devices (CSTDs) enable “safe” preparation of agents for medical personnel and ensure a safe hospital environment. However, artificial particles of infusion materials have been reported during CSTD use. Here, the incidence of insoluble fine particles during preparation of anticancer agents using CSTDs was examined. Visible insoluble fine particles were found in 465 (9.4%) of 4948 treatment cases at Ehime University Hospital with CSTD use. Contaminants occurred more frequently during preparation of monoclonal antibodies than cytotoxic anticancer agents (19.4% vs. 4.1%, respectively, *P* < 0.01). A similar survey was conducted at nine hospitals to investigate the incidence of insoluble fine particles with or without CSTDs. Insoluble fine particles were detected in 113 (15.4%) of 732 treatment cases during preparation of monoclonal antibodies with CSTD use. In contrast, the occurrence of insoluble fine particles without CSTDs was found in only 3 (0.073%) of 4113 treatment cases. Contamination with CSTDs might cause harmful effects on patients during cancer therapy. We strongly recommend the use of in-line filters combined with infusion routes after CSTD use to avoid contamination-associated adverse events.

## Introduction

Intravenous injectables consist of solution-containing therapeutic agents. However, endogenous artificial foreign substances may be introduced during several steps of manufacturing procedures by contaminated equipment or environmental sources. These foreign substances are designated as “insoluble foreign matters” or “insoluble fine particles”^1^. Insoluble foreign matters and insoluble fine particles are distinguished by size. Insoluble foreign matters are recognized as visible foreign contaminants of solutions that can be easily removed from materials. In contrast, very fine substances recognized with careful observation and scientific analysis are designated as insoluble fine particles. These fine particles are sometimes difficult to remove from materials during medical procedures, and may consequently cause biological effects on patient health outside of the original medical treatment. For example, intravenous administration of insoluble fine particles and glass ampoules has been noted to cause damage to the vein, lung, liver, and spleen in rare cases.^2–8^ Therefore, insoluble fine particles are potential health hazards and should be carefully removed from medical procedures in patients receiving long-term treatment, such as cancer patients.

In recent cancer treatment, occupational exposure of anticancer agents during preparation has been recognized as a serious environmental problem of cancer treatment^9–17^. Therefore, closed system drug transfer devices (CSTDs) were universally introduced to chemotherapy^18–22^. These technological improvements and devices have enabled safe administration of anticancer agents not only for patients and medical personnel but also hospital environments. However, CSTDs contain multiple moving parts such as vapor-trapping pockets with needles or plastic spikes, syringe units, and lubricants. Therefore, CSTD use may increase contamination of intravenous injectables and consequently be a source of health hazards in cancer patients receiving long-term medical treatment. Here, we analyzed the frequency and components of insoluble fine particles generated during the preparation of anticancer agents using CSTDs in infusion devices and discussed the influence, prevention, and countermeasures for cancer treatment.

## Results

### Incidence of insoluble fine particles generated during preparation of anticancer agents with CSTDs

**Figure 1:**
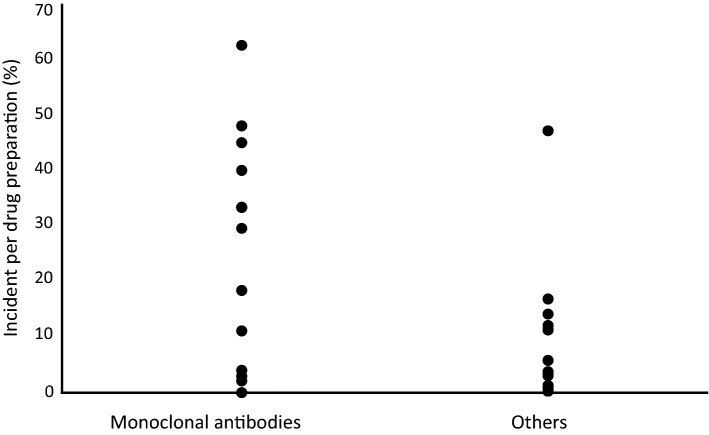
Frequencies of insoluble fine particles in preparation of monoclonal antibodies compared to those during preparation of cytotoxic agents at Ehime University Hospital. Others: all anticancer agents excluding monoclonal antibodies.

In early 2018, some pharmacists noticed the existence of insoluble fine particles generated during preparation of anticancer agents with CSTDs. After checking for these contaminants, we examined the frequency of insoluble fine particles during preparation of each anticancer agent for 1 year (January 31, 2018–January 30, 2019). Table 1 shows the incidence of insoluble fine particles by drug type (monoclonal antibodies versus cytotoxic agents) because we observed that the incidence of insoluble fine particles during preparation of recently produced monoclonal antibodies appeared to be higher than that during the preparation of cytotoxic agents. Pembrolizumab (anti-PD-1 antibody) showed the highest incidence of contamination of insoluble fine particles, accounting for 62.5% of contaminants. Other monoclonal antibodies, such as ramucirumab (48%), panitumumab (45%), and mogamulizumab (40%), also showed surprisingly higher incidents of insoluble fine particles compared to cytotoxic agents for cancer chemotherapies (Fig. 1).

**Table 1:** Incidence of insoluble fine particles during preparation of anticancer agents at Ehime University Hospital (frequency = incident/preparation)**.**

Agent	Incident (n)	Frequency (n)	Incident/frequency (%)
**Monoclonal antibodies**			
Pembrolizumab	70	112	62.5
Ramucirumab	36	75	48
Panitumumab	18	40	45
Mogamulizumab	2	5	40
Durvalumab	3	9	33.3
Daratumumab	19	57	33.3
Ipilimumab	1	3	33.3
Cetuximab	26	88	29.5
Bevacizumab	140	763	18.3
Elotuzumab	6	54	11.1
Brentuximab vedotin	1	25	4
Pertuzumab	3	89	3.4
Nivolumab	1	34	2.9
Rituximab	7	330	2.1
Aflibercept	0	5	0
Obinutuzumab	0	4	0
Atezolizumab	0	25	0
Total	333	1718	19.4
**Others**			
Pemetrexed	16	34	47.1
Pralatrexate	8	17	47.1
Vinorelbine (Navelbine^®^)	1	6	16.7
Eribulin	11	79	13.9
Cyclophosphamide	32	271	11.8
Paclitaxel (NK)	43	390	11
Carfilzomib	1	18	5.6
Nab-paclitaxel	9	257	3.5
Bendamustine	2	72	2.8
Irinotecan (Campto^®^)	2	205	1.0
Gemcitabine (Yakult)	4	435	0.9
Doxorubicin (NK)	1	214	0.5
Fluorouracil	2	483	0.4
Nedaplatin	0	2	0
Vinblastine	0	53	0
Oxaliplatin	0	262	0
Vincristine	0	123	0
Amrubicin	0	3	0
Carboplatin (NK)	0	170	0
Cisplatin (MARUKO)	0	88	0
Dacarbazine	0	22	0
Liposomal doxorubicin	0	10	0
Pirarubicin	0	16	0
Total	132	3230	4.1

### Occurrence of insoluble fine particles with and without CSTD use in other institutes

Eight hospitals including our institute and one cancer center in Ehime Prefecture participated in the current study. Among these institutes, three institutes routinely used CSTDs for all anticancer drugs. Two institutes used CSTDs only for highly carcinogenic anticancer agents, and the remaining four institutes used CSTDs only for volatile anticancer agents (cyclophosphamide, ifosfamide, and bendamustine). The summary of the preparation of anticancer agents with or without CSTDs in the nine institutes is indicated in Table 2.

**Table 2:** Summary of frequencies of contaminations at institutes providing chemotherapies in Ehime Prefecture.

Information of preparation	Hospital								
	Ehime Univ Hop	A	B	C	D	E	F	G	H
CSTD type	A	A	A	A	B	B	B	C	C
Preparations (n)	1818	4920	669	5701	158	585	205	582	2677
Frequencies of CSTD use/total chemotherapies (%)	81.6	5.2	100	14.8	88.9	4	14.3	6.9	6.4
Incidence of contaminations (n)	167	2	1	9	0	0	0	0	16
Frequencies of contaminations (total) (%)	9.19	0.041	0.15	0.16	0	0	0	0	0.6
Frequencies of contaminations (CSTD use) (%)	9.19	0	0.15	0.14	0	0	0	0	0
Frequencies of contaminations (needle preparation) (%)	0	0.041	–	0.02	0	0	0	0	0.6

Table 3 summarizes the frequencies of visible contamination of insoluble fine particles. Insoluble fine particles were found in 19 (0.14%) of 13,547 preparations without CSTD use. In contrast, insoluble fine particles were found in 176 (4.7%) of 3768 preparations with CSTD use. The incidence of visible contamination was significantly higher with CSTDs than without CSTDs (*P* < 0.01). These cases were examined by 104 pharmacists from all institutes included in the study to confirm the data (Table S1).

**Table 3:** Incidence and frequency of contaminations during preparation of anticancer agents at institutes in Ehime Prefecture providing chemotherapies with or without CSTD use.

Agent	CSTD use					
	Yes			No		
	Incident (n)	Frequency (n)	Incident/Frequency (%)	Incident (n)	Frequency (n)	Incident/frequency (%)
**Monoclonal antibodies**						
Aflibercept	3	3	100	0	44	0
Atezolizumab	0	4	0	0	38	0
Bevacizumab	26	273	9.5	0	994	0
Blinatumomab	0	0	–	0	20	0
Brentuximab Vedotin	0	9	0	0	6	0
Cetuximab	5	13	38.5	0	101	0
Daratumumab	6	17	35.3	1	97	1
Durvalumab	2	13	15.4	0	161	0
Elotuzumab	4	6	66.7	0	23	0
Trastuzumab	0	20	0	2	782	0.3
Ipilimumab	0	4	0	0	0	–
Mogamulizumab	2	6	33.3	0	4	0
Nivolumab	1	107	0.9	0	442	0
Obinutuzumab	0	15	0	0	40	0
Ofatumumab	0	0	–	0	3	0
Panitumumab	4	15	26.7	0	207	0
Pembrolizumab	41	78	52.6	0	323	0
Pertuzumab	0	25	0	0	239	0
Ramucirumab	14	62	22.6	0	231	0
Rituximab	4	58	6.9	0	124	0
Rituximab (KHK)	1	4	25	0	170	0
Trastuzumab Emtansine	0	0	–	0	64	0
Total	113	732	15.4	3	4113	0.073
**Others**						
Aclarubicin	0	0	–	0	17	0
Amrubicin	0	6	0	0	126	0
Azacitidine	0	0	–	0	323	0
Bendamustine	1	139	0.7	0	0	–
Bleomycin	0	0	–	0	32	0
Bortezomib	0	0	–	0	313	0
Busulfan	0	0	–	0	4	0
Cabazitaxel	0	0	–	0	49	0
Carboplatin	0	5	0	0	0	–
Carboplatin (NK)	0	374	0	0	262	0
Carfilzomib	0	0	–	0	65	0
Cisplatin (MARUKO)	1	53	1.9	0	381	0
Cisplatin (Nichi-iko)	0	13	0	0	138	0
Cyclophosphamide	10	534	1.9	0	0	–
Cytarabine	0	0	–	0	159	0
Cytarabine (TEVA)	0	0	–	2	64	3.1
Dacarbazine	0	14	0	0	30	0
Daunorubicin	0	0	–	0	9	0
Degarelix	0	0	–	0	69	0
Docetaxel (EE)	0	32	0	1	426	0.2
Docetaxel (Nipro)	0	0	–	0	61	0
Doxorubicin	0	8	0	0	9	0
Doxorubicin (NK)	0	139	0	0	93	0
Doxorubicin (sandoz)	0	0	–	0	23	0
Epirubicin RTU	0	0	–	0	3	0
Epirubicin (NK)	0	31	0	0	106	0
Eribulin	5	34	14.7	0	151	0
Etoposide (Lastet^®^)	0	9	0	0	20	0
Etoposide (sandoz)	3	165	1.8	0	80	0
Etoposide (Teva Takeda)	0	0	–	0	110	0
Fludarabine	0	0	–	0	11	0
Fluorouracil	0	102	0	0	0	–
Fluorouracil (TOWA)	0	172	0	0	1496	0
Gemcitabine	0	67	0	0	0	–
Gemcitabine (Hospira)	0	0	–	0	218	0
Gemcitabine (sandoz)	0	0	–	0	26	0
Gemcitabine (Yakult)	2	125	1.6	0	849	0
Idarubicin	0	0	–	0	5	0
Ifomide	2	39	5.1	0	15	0
Immunobladder^®^	0	0	–	0	6	0
Irinotecan (Campto^®^)	0	123	0	0	0	–
Irinotecan (Topotecin^®^)	0	0	–	0	44	0
Irinotecan (Hospira)	0	0	–	3	403	0.7
Irinotecan (sawai)	0	22	0	0	0	–
Irinotecan (Taiho)	0	0	–	0	277	0
L-Asparaginase	0	0	–	0	13	0
Liposomal Doxorubicin	0	3	0	0	23	0
Melphalan	0	0	–	0	4	0
Methotrexate	0	0	–	0	47	0
MitomycinC	0	0	–	0	15	0
Nab-paclitaxel	0	190	0	0	776	0
Nedaplatin	0	3	0	0	12	0
Nogitecan	0	3	0	0	27	0
Oxaliplatin	0	281	0	0	0	–
Oxaliplatin (Hospira)	0	18	0	10	391	2.6
Oxaliplatin (NK)	0	0	–	0	52	0
Oxaliplatin (sawai)	0	65	0	0	31	0
Paclitaxel	0	4	0	0	0	–
Paclitaxel (Hospira)	0	59	0	0	749	0
Paclitaxel (NK)	27	114	23.7	0	261	0
Pemetrexed	8	22	36.4	0	220	0
Picibanil^®^	0	0	–	0	4	0
Pirarubicin (Therarubicin^®^)	0	0	–	0	53	0
Pralatrexate	3	6	50.0	0	0	–
Ranimustine	0	0	–	0	2	0
Streptozocin	0	0	–	0	12	0
Vinblastine	0	17	0	0	31	0
Vincristine	0	32	0	0	106	0
Vindesin	0	0	–	0	3	0
Vinorelbine (Navelbine^®^)	1	13	7.7	0	47	0
Vinorelbine (Rozeus^®^)	0	0	–	0	82	0
Total	63	3036	2.1	16	9434	0.17

### Verification of insoluble fine particles using SEM, FTIR, and SEM-EDS analysis

**Figure 2:**
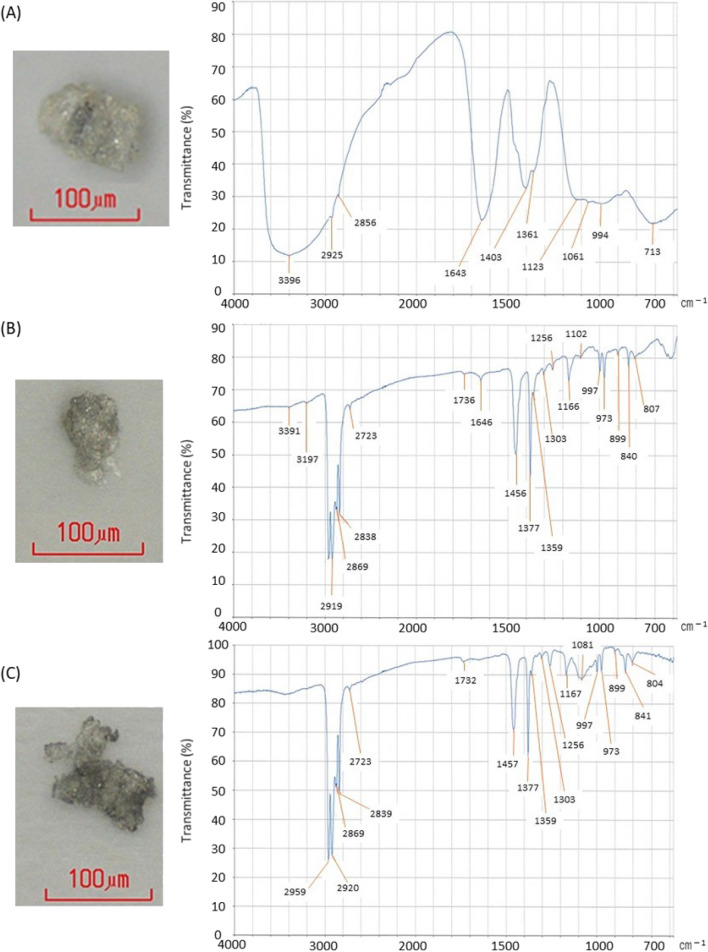
Visual and structural analysis of insoluble fine particles using microscopy and Fourier transform-infrared spectroscopy (FTIR). (**A**) A polysaccharide containing a carboxylic acid structure was considered as a contaminant by FTIR analysis. (**B**) FTIR analysis indicated that polypropylene was considered as the main component, and fatty acids were considered as the secondary component. (**C**) FTIR analysis indicated that the main component was polypropylene, and the possibility of silicone as an accessory component was considered.

To verify the number of insoluble fine particles trapped in in-line filters after cancer chemotherapies, 13 in-line filters and 3 control filters were randomly selected and recovered. Insoluble fine particles trapped in in-line filters were observed with a microscope, and trapped components were analyzed by FTIR. Figure 2 shows representative photographs of insoluble fine particles, gray substances measuring approximately 100 μm, trapped in the in-line filter. FTIR analysis indicated that one gray particle (Fig. 2A) was a polysaccharide containing a carboxylic acid structure. Another gray particle (Fig. 2B) consisted of polypropylene as the main component, suggesting that this was a fatty acid component. The structure of the last particle (Fig. 2C) seemed to be polypropylene, suggesting that this was silicone as an accessory component in CSTDs. From these analyses, we decided to implement routine use of in-line filters into infusion routes to avoid biological damage from insoluble fine particles during cancer treatment. The possibility of other contaminants and debris, such as rubbers, plastic utensils, and needle shards, could not be completely denied from past reports^23–25^.

## Discussion

Our current study detected three important findings. First, the incidence of insoluble fine particles in cases using CSTDs during cancer chemotherapies was significantly higher than in those not using CSTDs. Second, the incidence of insoluble fine particles was significantly higher in cases treated with monoclonal antibodies than in those treated with other cytotoxic anticancer agents. Third, the incidence of insoluble fine articles varies among medical institutes.

The risk of contamination by insoluble fine particles in injection materials has been noted and was discussed in the early 1960s^23^. The British Pharmacopoeia subsequently announced and established regulations on insoluble fine particles in injection materials in 1973, and the United States Pharmacopeia also established similar regulations in 1984^26,27^. The criteria for insoluble fine particles in injectables have been described in the 14th revised Japanese Pharmacopoeia in 2001 to ensure the safety of injectables in Japan^28^. Consequently, contamination with insoluble fine particles during manufacturing procedures were found to be within the standard range. However, with recent advances in cancer chemotherapies, several materials are used in the preparation and administration of drugs used during the treatments, which require complicated management of each agent, such as storage, dispensing and administration. One of the complexities is derived from CSTD-associated materials. A CSTD can prevent biohazards. However, manufacture and use of CSTDs involves several steps and related materials, such as vapor-trapping pockets, needles or plastic spikes, syringe units and lubricants. Our multicenter study also showed that 180 insoluble fine particles out of the 195 cases of contamination were found especially in mixing syringes (data not shown) during the process of preparation, suggesting that the insoluble fine particles might be derived from certain chemical reactions or from aggregations of proteins sheared during mixing, as other possible contaminants. Our current observation suggests that CSTD use might increase the number of contaminants and their aggregation in intravenous injectables, subsequently augmenting the risk of health hazards during cancer chemotherapies.

Interestingly, we detected a higher incidence of insoluble fine particles in patients treated with monoclonal antibodies. Nakayama et al.^29^ reported several differences in solutions, coating materials of vials, and type (material and hardness) of rubber stoppers used as recent medical materials. In the current study, insoluble fine particles were more frequently generated in cases using CSTDs than in cases using conventional preparations (syringe and needle) without CSTDs. For example, a coating material such as silicone oil is applied to the inside of the syringe to improve slidability of the plunger. For low molecular weight compounds, application of silicone oil is not a major problem; however, for protein preparations, such as monoclonal antibodies application of silicone oil might cause protein aggregation^30,31^. We speculate that this might explain the high incidence of contamination in cases treated with monoclonal antibodies. On the other hand, immunogenicity in the human body after inadvertent injection of the aggregates formed by the reaction of silicone oil and proteins, including monoclonal antibodies, is not fully understood^32–34^.

We also noted differences in the incidence of contaminants among medical institutes. Since the first observation of insoluble fine particles during preparation of anticancer agents in early 2018, pharmacists at Ehime University Hospital have carefully checked for impurities. However, the incidence of contamination at our institute in this survey was extremely high (9.2%; 167 CSTD cases of 1818 preparations of anticancer agents at Ehime University Hospital) compared to those of other institutes (0.4%; 9 CSTD cases of 2227 preparations of anticancer agents in 7 other institutes) (Table 3). This discrepancy may be partially explained by the number of patients receiving monoclonal antibody treatments and types of material used, such as CSTD, infusion route, needle, and syringe. However, we could not determine the precise reason for this difference in incidence.

Lastly, our analysis suggested that the possible contaminants among insoluble fine particles might include polysaccharides, fatty acid components and silicone, which are probably derived from the coating materials of vials, syringes, and CSTDs and related materials. However, the number of samples examined by FTIR and SEM-EDS was limited. Hence, the possibility of other contaminants and debris, such as rubber, plastic utensils and needle shards, could not be completely denied. Furthermore, we observed oleamide crystals in infusion tubes (data not shown). Oleamide is used as a coating material for intravenous tubing and has no adverse effects in humans. However, the effect of long-term exposure to oleamide crystals in patients, especially cancer patients, is not fully understood, suggesting that it might be better to avoid this contaminant as much as possible. Intravenous administration of insoluble fine particles and glass ampoules has been noted to cause damage to the vein, lung, liver and spleen in rare cases^2–8^. From past reports^30,31^ and the current study showing an increase in insoluble fine particles during monoclonal antibody preparation, other possible aggregates formed by the reaction of silicone oil and monoclonal antibodies or due to protein shearing should also be considered. When insoluble fine particles are detected in the preparation of anticancer agents using CSTDs, it might be impossible to identify the nature of the foreign substance (glass amplifiers, rubber, plastic utensils, needle shards, protein aggregates, etc.). Therefore, we strongly recommend the use of in-line filters to prevent the intravenous injection of artificial contaminants in anticancer agents or monoclonal antibodies administered with CSTDs. Cancer treatments typically induce adverse events such as allergy, chills and fever. However, some of these adverse events might be due to artificial contaminants during CSTD use, which can be prevented by the routine application of in-line filters.

## Materials and methods

### Incidence of insoluble fine particles generated during preparation of anticancer agents using CSTDs at Ehime University Hospital

From January 31, 2018 to January 30, 2019 (1-year period), the frequency of insoluble fine particles discovered in the chemotherapy room for outpatients at Ehime University Hospital was aggregated by each anticancer agent. Insoluble fine particles were examined during preparation procedures for anticancer agents, such as in syringes adjusted with anticancer agents, vials after mixture, and infusion bags after CSTD use. Once insoluble fine particles were recognized at any step of the preparation procedure, an additional pharmacist confirmed the presence of insoluble fine particles. When multiple insoluble fine particles were observed during different preparation steps of one anticancer agent, the number of insoluble fine particles generated was counted as one. The incidence of insoluble fine particles divided by number of drug preparations, i.e., frequency/anticancer agent, was aggregated and statistically analyzed.

### Comparison of frequency of insoluble fine particles with and without CSTD use at other hospitals in Ehime Prefecture

Frequencies of drug contamination by insoluble fine particles at eight hospitals, including Ehime University Hospital and one cancer center, were investigated using the same method. We created a video explaining the preparation steps, their processes, and confirmation points on YouTube (https://youtu.be/RRy6mqgfYmc), and started the analysis after viewing the video. Each anticancer agent was diluted in a 50–1000 mL infusion bag based on the manufacturer’s recommendations. The infusion bags were all transparent bags made of polyethylene, polypropylene or polyethylene–vinyl acetate copolymer. The presence of insoluble fine particles was examined using a safety cabinet with a silver-colored background under cabinet light with an intensity of 1000 lux or more. If insoluble fine particles were recognized at any step of the preparation procedure, their presence was confirmed by an additional pharmacist (double-checked by two pharmacists). Each institute evaluated the frequency of contamination over a consecutive 3-month period between January and June 2019, and the incidence of contamination of insoluble particles and CSTD types were compared.

### Verification of insoluble fine particles after CSTD use

To avoid hazardous effects in cancer patients receiving chemotherapy after observation of insoluble fine particles with CSTD use, we applied in-line filters (0.2 μm) to trap the impurities during chemotherapy starting from March 2018 in our institute. Therefore, we next attempted to verify insoluble particles to analyze the trapped materials.

After chemotherapy, in-line filters were randomly selected, recovered, and washed five times with distilled water, and rinses were collected. Filter membranes were then dried, insoluble fine particles trapped within the in-line filter were observed using a microscope, and trapped components were analyzed by Fourier transform-infrared spectroscopy (FTIR) (Spotlight 400, PerkinElmer Inc., Wellesley, MA, USA). As controls, in-line filters without CSTD use were collected after saline infusion. Additionally, materials such as rubber stoppers of anticancer drug vials, membranes of the connection part in CSTDs, and needles stored in CSTDs were analyzed as possible sources of contaminants by FTIR and scanning electron microscopy-energy dispersive X-ray spectroscopy (SEM-EDS) (SEM: Quanta 200 FEG, FEI Company, Hillsboro, OR, USA; EDS: INCA Energy, OXFORD Inc., Oxford, UK) to clarify the insoluble fine particles trapped in in-line filters.

### Statistical analysis

Statistical analyses were performed using SAS software package version 9.4 (SAS Institute Inc., Cary, NC, USA). Fisher’s exact test was used for categorical data. *P* values < 0.05 were considered indicative of statistical significance.

### Ethics of the study

This study was approved by the Ethics Committee for Clinical Studies at Ehime University Graduate School of Medicine (study #1810019) and was carried out in accordance with the ethical standards of the 1995 Declaration of Helsinki (as revised in Brazil, 2013). Opt-out principle was conducted in this study, and the documents were shown in https://www.m.ehime-u.ac.jp/school/clinical.oncology/?page_id=25. Informed consent was obtained from all participants and/or their legal guardians.

## Supplementary Information

Supplementary Table S1.
