# Supplementary material for: Frequency and component analysis of contaminants generated in preparation of anticancer agents using closed system drug transfer devices (CSTDs)
Source: Sci Rep. 2022 Jan 7;12:139. doi: 10.1038/s41598-021-03780-0 (PMC8741972; doi:10.1038/s41598-021-03780-0)
Supplement: Supplementary file 1 — Supplementary Table S1. [file 41598_2021_3780_MOESM1_ESM.docx]

**Frequency and component analysis of contaminants generated in preparation of anticancer agents using Closed system drug transfer devices (CSTDs)**

Satomi Sumikawa^1^, Yoshihiro Yakushijin^2*^, Kenjiro Aogi^3^, Takuya Yano^4^, Hiroki Hashimoto^5^, Chiyuki Tsukui^6^, Tadashi Noguchi^7^, Taro Shiraishi^8^, Yasuhiro Horikawa^9^, Yasuo Yasuoka^10^, Akihiro Tanaka^11^, Noriaki Hidaka^1^ and Mamoru Tanaka^1^

**Supporting information**

**Table S1.** Information on pharmacists engaged in the preparation of anticancer agents.
